# Why national health research systems matter

**DOI:** 10.1186/1478-4505-6-1

**Published:** 2008-01-11

**Authors:** Stephen R Hanney, Miguel A González Block

**Affiliations:** 1 Health Economics Research Group, Brunel University, Uxbridge, UK; 2 Center for Health Systems Research, National Institute of Public Health, Cuernavaca, Mexico

## Editorial

How to organise health research systems so as to maximise the benefits is increasingly debated at the national and international level, with some highly innovative developments resulting. We intend to publish a series of articles in *Health Research Policy and Systems *(HARPS) that describe and analyse these developments in various ways; both so that lessons might be passed on and to stimulate similar debates and commentaries in other countries. Adopting the perspective of a national system for health research immediately raises some perspectives with which a strand of traditional academic thinking is uncomfortable. And yet, for example, whilst the position of the National Institutes of Health in the USA is unrivalled in terms of the amount of high quality biomedical science it produces, questions are being asked as to why the health care system is ranked below that of many other developed countries in recent assessments [1]. Is there something lacking in the health research system in the USA at the overall level that contributes to this apparent paradox, or is it caused solely by factors within the American health care, political and socio-economic systems?

Many researchers believe they are most likely to make progress when they are funded (admittedly usually from the public purse) to pursue the topics that arise from within science [2] or, in the case of some medical academics, from the interplay of science and unresolved issues thrown up by their clinical practice. Serendipity is seen as being essential, with the story of Alexander Fleming's 'accidental' discovery of penicillin frequently told. Some authors even claim public funding for research, however it is organised, is inherently inefficient [3]. At the same time, public authorities in many countries are increasingly both funding research and interested in what comes from their funding.

In our previous editorial in HARPS we addressed some of the many specific aspects that make up the discussions around health research systems [4]. Here we revisit some of these issues, but concentrate on analysis at the level of systems and how they are best organised. There has long been a desire from some for a greater degree of intervention over how the research system funded by the state is organised. The debate becomes even more complex when researchers who study scientific systems argue that research increasingly is, and should be, undertaken in a 'context of application' [5]. Margaret Thatcher, as Prime Minister, dominated British politics throughout the 1980s and was famed for showing few doubts about the policies she was pursuing, but during her period intense but uncertain debates raged as to how far academics should be free to pursue their own priorities [6,7].

What is needed, we submit, is detailed analysis, and action, at the national (and international?) level that adopts a systems approach. This does not necessarily mean the state or its proxies should be deeply involved in controlling all aspects of the system; it might well be best if within the system there are diverse approaches each geared to meeting different needs. But the issues need to be considered at an overall level.

UK and Canada provide interesting examples of how some of the key debates have played out. There have been detailed accounts of initiatives in the UK to make the system more responsive to the needs of the health care system at particular times [8-10].

In HARPS we shall be publishing a number of articles that in different ways consider developments in the organisation of UK and Canadian health research systems. We will publish an account [11] that analyses the twists and turns in the UK health research system over almost a century and reveals some surprisingly early interventionist themes that challenge some widely held perceptions about the universality of the freedom offered to researchers. Crucially the authors demonstrate that the key problem was less one of fundamental philosophical objections to some health research being responsive to the needs identified by the health care system, but rather practical difficulties in organising such a system: 'vision was not matched by means' [11]. One of the benefits of adopting such a detailed historical approach is that developing an understanding of how the debates have played out over many decades should help inform current thinking.

Undeniably in the UK, as probably elsewhere, there has been an increase in the number of stakeholders with an interest in how the health research system is organised. Currently there is a fast evolving attempt to create an overall system that builds on reforms over the last 30 years and meets the needs of various stakeholders. We hope to publish a study that adopts a systems approach to analyse how far these various needs can be accommodated.

In Canada a major reform of the health research system in 2000 resulted in the creation of the Canadian Institutes of Health Research, which has been widely admired. It explicitly describes its results as being, 'improved health for Canadians, more effective health services and products, and a strengthened Canadian health care system' [12]. As part of the overall reforms of health research in Canada, and as highlighted in our previous editorial, the Canadian Health Services Research Foundation (CHSRF) is conducting innovation, evaluation and analysis into ways of organising health research aimed at ensuring that part of the system meets needs of the policy-makers. These developments were spearheaded by the first Director of CHSRF, Jonathan Lomas, with his concept of 'linkage and exchange' [13]. They have generated considerable international attention. Following Lomas's recent retirement we have commissioned an appreciation of his work that should be featured early in 2008.

What unites these forthcoming papers in HARPS is the focus on analysis of the ways health research systems can best be organised to improve health care. This is central to the mission of HARPS.

## References

[B1] World Health Organization (2000). The World Health Report 2000 – Health Systems: Improving Performance Geneva.

[B2] Polanyi M (1962). The Republic of Science: its political and economic theory. Minerva.

[B3] Kealey T (1996). The Economic Laws of Scientific Research.

[B4] Hanney S, González Block MA (2006). Building health research systems to achieve better health. Health Res Policy Syst.

[B5] Gibbons M, Limoges C, Nowotny H, Schwartzman S, Scott P, Trow M (1994). The New Production of Knowledge: The Dynamics of Science and Research in Contemporary Societies.

[B6] Thatcher M (1993). The Downing Street Years.

[B7] Edgerton D, Hughes K (1989). The poverty of science: a critical analysis of scientific and industrial policy under Mrs Thatcher. Public Administration.

[B8] Kogan M, Henkel M (1983). Government and Research: The Rothschild Experiment in a Government Department.

[B9] Black N (1997). A national strategy for Research and Development: Lessons from England. Annu Rev Public Health.

[B10] Kogan M, Henkel M, Hanney S (2006). Government and Research: Thirty Years of Evolution.

[B11] Shergold M, Grant J Freedom and need: the public strategy for biomedical and health research in England. Health Res Policy Syst Forthcoming.

[B12] Canadian Institutes for Health Research Who we are. http://www.cihr.ca/e/7263.html.

[B13] Lomas J (2000). Using ‘linkage and exchange’ to move research into policy at a Canadian Foundation.. Health Aff.

